# Supplementary material for: Growth and behaviour of blue mussels, a re-emerging polar resident, follow a strong annual rhythm shaped by the extreme high Arctic light regime
Source: R Soc Open Sci. 2020 Oct 14;7(10):200889. doi: 10.1098/rsos.200889 (PMC7657935; doi:10.1098/rsos.200889)
Supplement: Table S1. Timetable of the study [file rsos200889supp1.pdf]

Table S1. Timetable of the study. Dates of the different studied periods during the experience lasting 712 days, from 5 May 2016 to 17 April 2018. In total 8 studied periods : 2 polar days; 2 polar nights; 2 periods with days with light / dark alternation centered on the spring equinox (Spring LD alternation); 2 periods with days with light / dark alternation centered on the autumn equinox (Autumnal LD alternation). 15 days of record are missing due to electrical failure.

Periods	**Polar day 1**	**Automnal LD alternation 1**	**Polar night 1**	**Spring LD Alternation 1**	**Polar day 2**	**Automnal LD alternation 2**	**Polar night 2**	**Spring LD alternation 2**
Dates	05/05/2016-26/08/2016 *114 days*	27/08/2016-23/10/2016 *58 days*	24/10/2016-16/02/2017 *116 days*	17/02/2017-16/04/2017 *59 days*	17/04/2017-26/08/2017 *132 days*	27/08/2017-24/10/2017 *59 days*	25/10/2017-17/02/2018 *116 days*	18/02/2018-17/04/2018 *60 days*
Missing data	_	_	26/12/2016-09/01/2017 *15 days*	_	_	_	_	_
