# Supplementary material for: Growth and behaviour of blue mussels, a re-emerging polar resident, follow a strong annual rhythm shaped by the extreme high Arctic light regime
Source: R Soc Open Sci. 2020 Oct 14;7(10):200889. doi: 10.1098/rsos.200889 (PMC7657935; doi:10.1098/rsos.200889)
Supplement: Figure S2. Individual double-plotted actogramms of valve opening amplitude (VOA) [file rsos200889supp3.pdf]

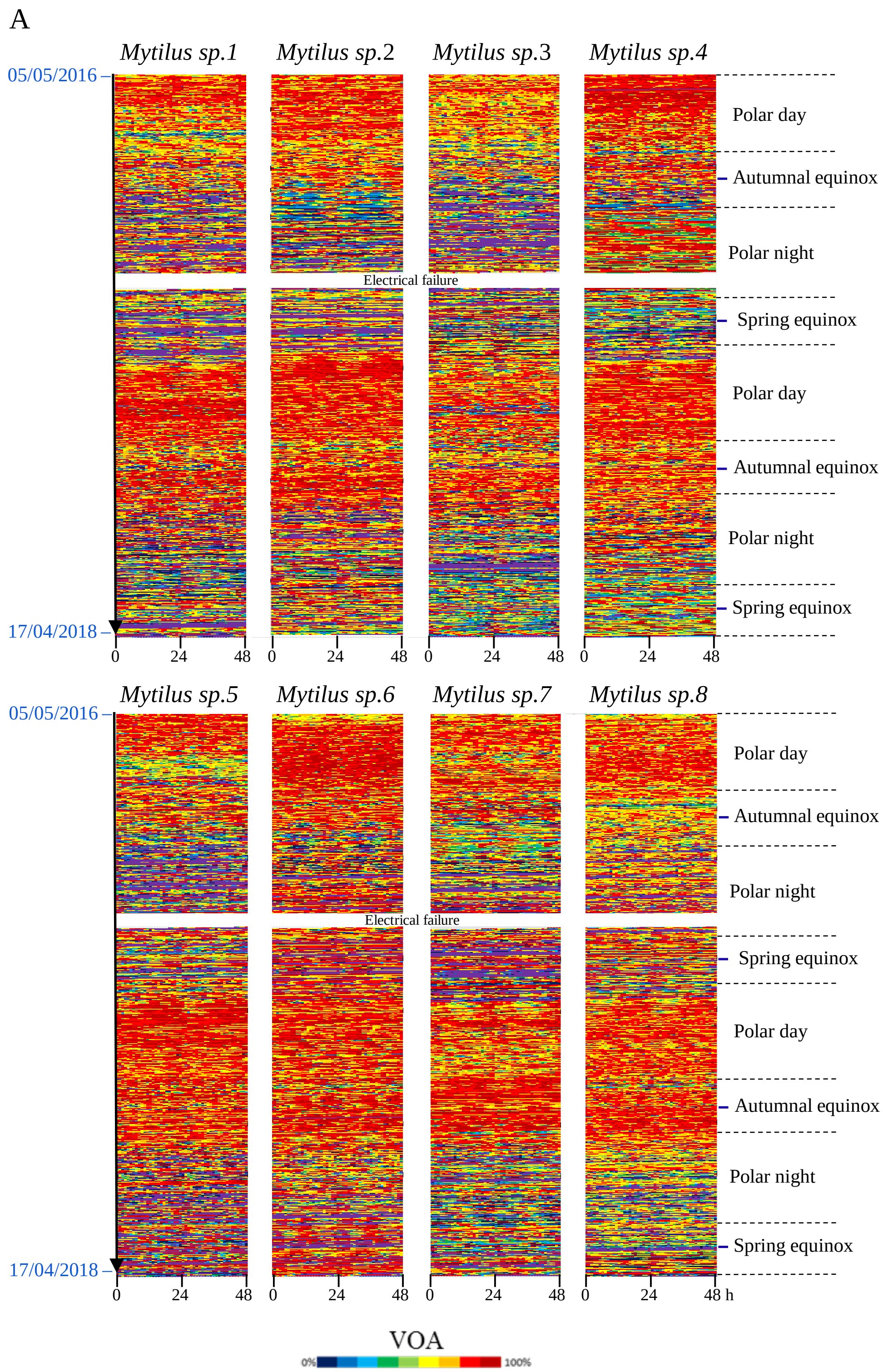
A
Mytilus sp.1
Mytilus sp.2
Mytilus sp.3
Mytilus sp.4
05/05/2016
17/04/2018
Polar day
Autumnal equinox
Polar night
Electrical failure
Spring equinox
Polar day
Autumnal equinox
Polar night
Spring equinox
0 24 48
Mytilus sp.5
Mytilus sp.6
Mytilus sp.7
Mytilus sp.8
05/05/2016
17/04/2018
Polar day
Autumnal equinox
Polar night
Electrical failure
Spring equinox
Polar day
Autumnal equinox
Polar night
Spring equinox
0 24 48 h
VOA
0%
100%

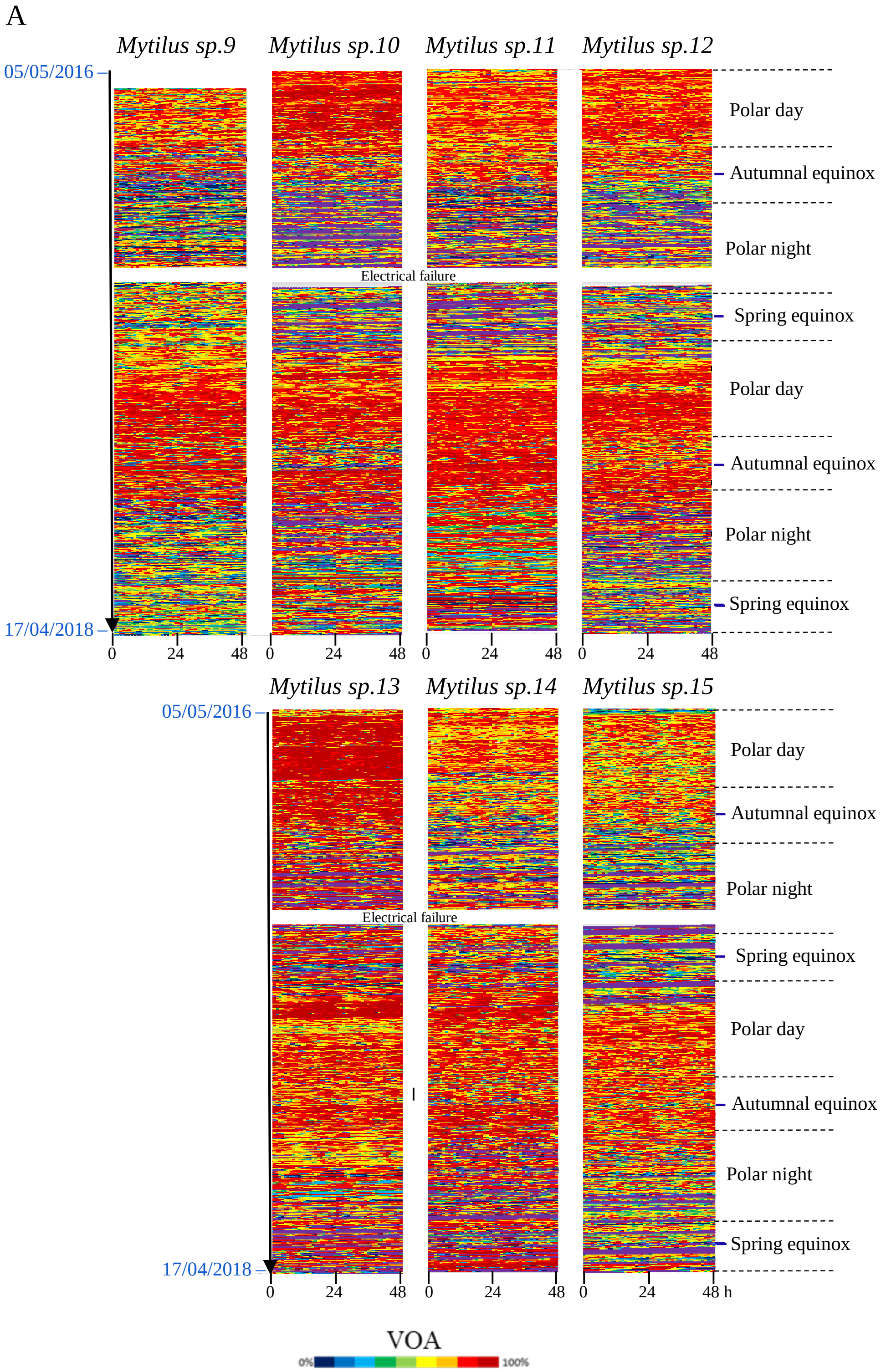
A
Mytilus sp.9
Mytilus sp.10
Mytilus sp.11
Mytilus sp.12
05/05/2016
17/04/2018
Polar day
Autumnal equinox
Polar night
Electrical failure
Spring equinox
Polar day
Autumnal equinox
Polar night
Spring equinox
0
24
48
Mytilus sp.13
Mytilus sp.14
Mytilus sp.15
05/05/2016
17/04/2018
Polar day
Autumnal equinox
Polar night
Electrical failure
Spring equinox
Polar day
Autumnal equinox
Polar night
Spring equinox
48 h
VOA
0%
100%

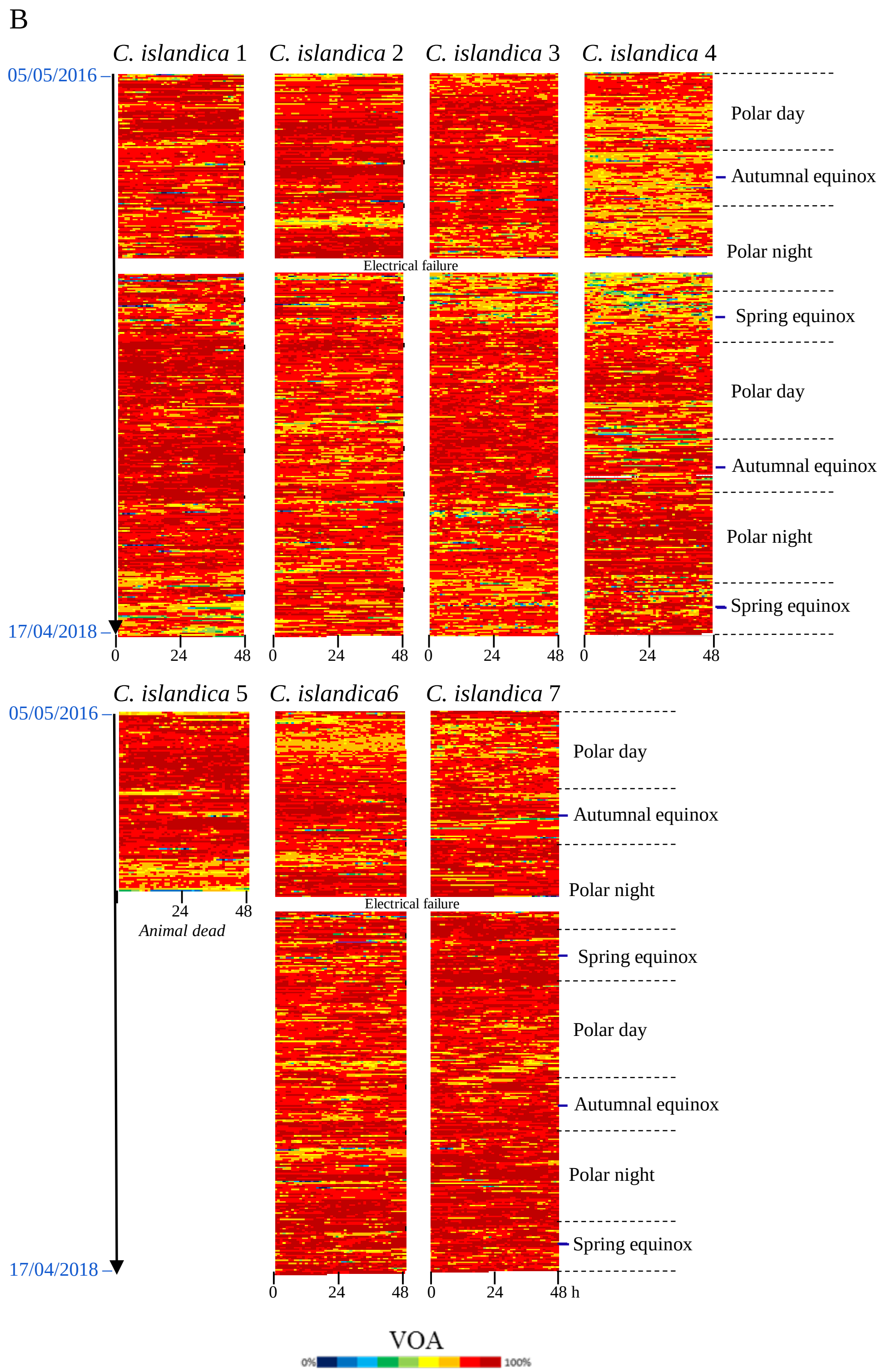

Figure S2. Individual double-plotted actogramms of valve opening amplitude (VOA) in the Kongsfjorden, Ny-Alesund, Svalbard recorded during 712 days (from 05/05/2016 to 17/04/2018) . (A) The blue mussels *Mytilus sp.* (n = 15) and (B) the scallops *C. islandica* (n = 7).
