# Supplementary material for: Growth and behaviour of blue mussels, a re-emerging polar resident, follow a strong annual rhythm shaped by the extreme high Arctic light regime
Source: R Soc Open Sci. 2020 Oct 14;7(10):200889. doi: 10.1098/rsos.200889 (PMC7657935; doi:10.1098/rsos.200889)
Supplement: Figure S3. Individual profiles of shell growth. [file rsos200889supp4.pdf]

Mytilus sp.

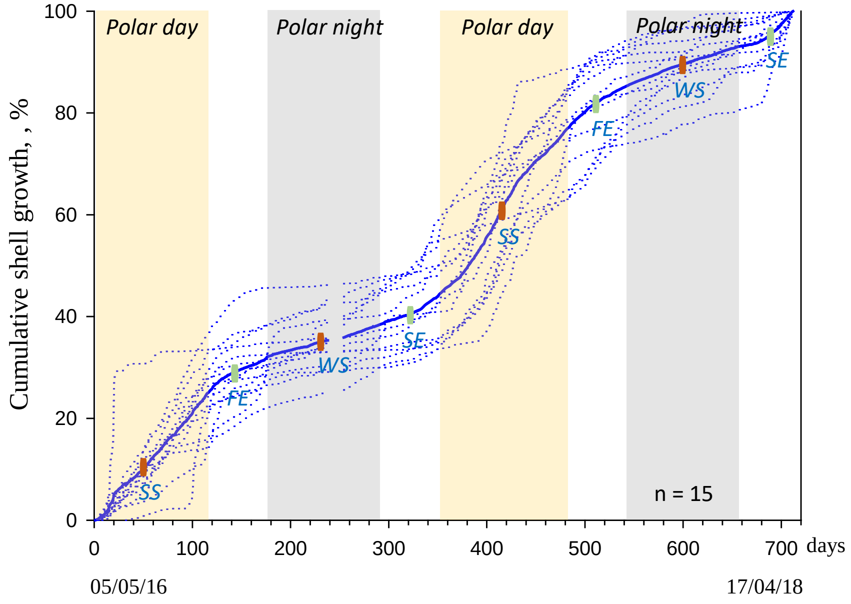

Chlamys islandica

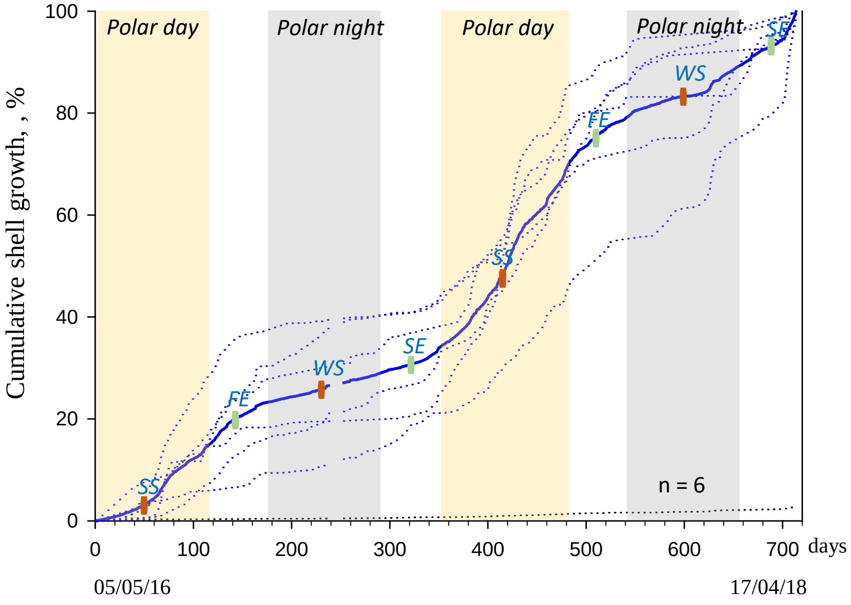

Figure S3. Individual profiles of shell growth. Upper panel and lower panel show the individual (doted lines) and mean (solid bold line) cumulative growth of the shells for *Mytilus sp*. (n = 15) and *C. islandica* (n = 6) respectively, during the 712-days of the in situ experiences for). SE: spring equinox; SS: summer solstice; AE: autumn equinox, WS: winter solstice.
