# Supplementary material for: Growth and behaviour of blue mussels, a re-emerging polar resident, follow a strong annual rhythm shaped by the extreme high Arctic light regime
Source: R Soc Open Sci. 2020 Oct 14;7(10):200889. doi: 10.1098/rsos.200889 (PMC7657935; doi:10.1098/rsos.200889)
Supplement: Table S4. Tables of Pearson's correlation coefficients. [file rsos200889supp5.pdf]

Table S4. Tables of Pearson's correlation coefficients. A: the scallop *C. islandica*, B: the mussel *Mytilus sp*. Each cell contents the correlation coefficient and the *p*-value in italic. Asterisks refer to significativity level with *: $p < 0,05$; **: $p < 0,01$; *** $p < 0,001$.

A

C. islandica	VOA	Growth	Photoperiod	Temperature
VOA		0.584<br>*0.129*	0.248<br>*0.554*	0.240<br>*0.605*
Growth			0.698<br>*0.0544*	0.672<br>*0.0981*
Photoperiod				0.781*<br>*0.0381*

B

Mytilus sp.	VOA	Growth	Photoperiod	Temperature
VOA		0.973***<br>*0.000046*	0.918**<br>*0.00130*	0.710<br>*0.0740*
Growth			0.941***<br>*0.000501*	0.775*<br>*0.0408*
Photoperiod				0.781*<br>*0.0381*
